# Current knowledge of scoliosis in physiotherapy students trained in the United Kingdom

**DOI:** 10.1186/s13013-017-0141-z

**Published:** 2017-09-27

**Authors:** D.A. Jason Black, Christine Pilcher, Shawn Drake, Erika Maude, David Glynn

**Affiliations:** 1 Scoliosis SOS Clinic, 63 Mansell Street, London, E1 8AN UK; 2 Arkansas State University, PO Box 910, Jonesboro, AR 72467 USA; 3 Independent, York, UK

**Keywords:** Scoliosis, Physiotherapy, University, Knowledge, Education, Treatment, Bracing, Causes, Screening, Diagnosis

## Abstract

**Background:**

It has been highlighted in both Poland and the United States of America (USA) that knowledge of idiopathic scoliosis (IS) among physiotherapy students is limited with respect to the 2011 International Society on Scoliosis Orthopaedic and Rehabilitation Treatment (SOSORT) guidelines. Early detection of scoliosis and correct initial management is essential in effective care, and thus physiotherapists should be aware of the basic criteria for diagnosis and indications for treatment. The aim of this study was to evaluate the basic knowledge of IS in physiotherapy students trained in the United Kingdom (UK).

**Methods:**

A previously designed and tested 10-question survey, including knowledge of the 2011 SOSORT guidelines, was transcribed onto an online-survey platform. Questions were designed to analyse knowledge of definition, cause, development, prevalence, diagnosis, treatment and bracing of scoliosis.

All UK universities offering physiotherapy degrees were invited to participate, with the programme lead of each institution asked to distribute the questionnaire to all penultimate and final year physiotherapy students (bachelor’s and master’s degrees). The final number of students who received the study invitation is unknown. The survey link closed after 8 weeks of data collection.

**Results:**

Two hundred and six students, split over 12 institutions, successfully completed the questionnaire.

Analysis showed that 79% of students recognised when IS is likely to develop, yet only 52% recognised that IS’s aetiology is unknown. Eighty-eight percent of students incorrectly defined IS as a 2-dimensional deformity, with only 24% successfully recognising the prevalence of IS within the scoliosis population. Just 12% knew the criteria for diagnosis; however, 93% were unable to recognise the appropriate treatment approach through therapeutic exercise. Finally, 54% of students managed to identify correctly when bracing is recommended for IS.

In comparison to previous studies within the USA, students in the UK performed worse in relation to all questions except treatment (7% answered correctly vs 3% in the American study).

**Conclusion:**

With only 7% of students able to answer > 50% of the survey questions correctly, there is a clear lack of knowledge of appropriate IS diagnosis and care which could directly impact the information these patients are given within the first contact primary care in the UK.

**Electronic supplementary material:**

The online version of this article (10.1186/s13013-017-0141-z) contains supplementary material, which is available to authorized users.

## Background

Scoliosis refers to a 3-dimensional deformity of the spine [1] and is a condition which is generally accepted as affecting 2–3% of children aged 12–16 years old [2]. The current knowledge of scoliosis, including its diagnosis and management among physiotherapy students’ in UK, is not known. A few previous studies completed outside the UK have investigated this, largely reporting a poor result.

Drake et al. designed a 10-question multiple choice survey to establish the knowledge of scoliosis diagnosis and treatment in 178 physiotherapy students in the USA. Results were poor, showing a mean overall correct score of 43%, with only 15 students answering over 70% correctly [3].

A study completed in Poland by Ciazynski et al. also tested the knowledge of physiotherapy students in this field using a similar questionnaire format to the study by Drake et al. [4]. Apart from the much smaller subject numbers in this study (37), the students had already covered conservative treatment methods for scoliosis in their syllabus. These students generally performed more favourably in comparison to those in the study by Drake et al. [3], noting scoliosis as a 3-dimensional deformity (81.3 versus 29%) and how to confirm diagnosis (62.2 versus 20%). Most participants (90.5%) in the study by Drake et al. [3] were not familiar with any conservative treatment methods, whereas most students (94.6%) were aware of at least one conservative treatment method in the study by Ciazynski et al. [4], who recommends that education for scoliosis among physiotherapy students should be comprehensive and cover the current SOSORT guidelines.

With an increase in self-referral to physiotherapy in the UK [5], the likelihood of a physiotherapist being the first point of contact for a patient with scoliosis is increased. For this reason, it could be considered particularly important that physiotherapists have the knowledge and understanding to be able to screen and recognise the symptoms of a patient with scoliosis effectively. Furthermore, it is crucial for physiotherapists to know when it is appropriate to refer on and what treatment options are available.

Few text books from the recommended reading lists for physiotherapy degree courses in the UK mention scoliosis and most universities do not cover scoliosis as part of their syllabus or recommend specific reading on this topic. Therefore, it is unlikely many physiotherapy students in the UK will have adequate knowledge to be able to recognise and effectively manage patients with scoliosis. No study has verified the knowledge of IS diagnosis and management in physiotherapy students in the UK; therefore, this study has chosen to establish this in students in their penultimate and final years of physiotherapy degrees across different universities in the UK.

## Objectives

The aim of this study is to analyse the current knowledge of physiotherapy students at a university within the UK on their understanding of IS referring to the 2011 SOSORT guidelines. Thereby, investigating their proficiency as physiotherapists to provide screening, advice and exercise prescription to self-referring patients with IS.

## Methods

### Questionnaire development

Development of a questionnaire was completed by Drake et al. [3] using a theoretical framework from a previously completed student survey by Cziazynski et al. [4] and utilising the information provided within the 2011 SOSORT guidelines [1]. The survey was split into two distinct sections: seven questions based on analysing knowledge of scoliosis (definition, cause, development, prevalence, diagnosis, treatment and bracing) and three multiple choice questions looking at the students’ opinions on what exercises they thought might be beneficial or detrimental to a patient’s scoliosis, as well as their familiarity with accepted physiotherapy treatment methods for scoliosis.

The initial survey drafts were reviewed by colleagues at the Department of Physical Therapy at Arkansas State University. Following this, the second draft was reviewed by a panel of content experts, consisting of physical therapists specialising in treatment of scoliosis.

Following validation of the initial survey, a pilot study was completed with 27 physical therapy students at Arkansas State University.

### Questionnaire distribution

Taking this validated and tested questionnaire, the 10-question survey [Additional file 1] was transcribed onto an online-survey platform. All UK universities offering physiotherapy degrees were invited to participate in the study through an introductory email to the programme lead of each institution. Supportive programme leads were asked to distribute the questionnaire to all students who met the inclusion criteria. The online questionnaire was accessible for a period of 8 weeks in the period of Autumn 2015, at which point the survey link was closed and all data were accumulated for analysis.

### Inclusion and exclusion

The inclusion criteria consisted of UK students who were in their penultimate or final year for either a bachelor’s or master’s physiotherapy degree. Excluded from the study were any students who had recently graduated or were unable to complete the entire questionnaire.

All students were initially asked for their consent to participate in the study and then requested to complete the survey in one independent sitting without the use of external resources. The information collected from the student was their initials, current degree and stage of degree completion, university attended and if they had any previous experience with scoliosis.

### Ethical approval

The need for ethical approval was waived by the Council for Allied Health Professions Research. Permission to distribute the survey was obtained from the the Subject/Clinical Lead at each institution that participated.

## Results

Following completion of the study period, 206 students had attempted the questionnaire and met the required inclusion criteria, with 165 completing the questionnaire fully and thus being included in all further analysis. The students were spread across 12 different universities in the UK, encompassing Scotland, England, Wales and Northern Ireland.

To remain in-line with the methods used by Drake et al. [3], and to enable a direct comparison between the results of these two studies, the 31 students that responded ‘I Do Not Know’ were removed from the analysis of that question. Therefore in order to allow comparison to previous research, the ‘% of Correct Responses’ when discussed within each item section below was the percentage of students who selected the commonly accepted answer to each question, posed against those who provided an attempted response and removing ‘I Do Not Know’ as an option (Column F). The table below for reference also has an analysis of those subjects who answered as ‘I Do Not Know’ as a demonstration of ignorance to the topic and a more true representation of correct responses for future research (Column E) (Table 1).

**Table 1:** Results of survey per question

Column A Question	Column B Number of answer attempted (%)	Column C Number of responses removing ‘I Do Not Know’	Column D Number of correct responses	Column E Percent of correct responses (excluding unanswered)	Column F Percent of correct responses (excluding unanswered and ‘I Do Not Know’)
1—Definition	190 (92%)	164	19	10	12
2—Cause	183 (89%)	137	71	39	52
3—Development	173 (84%)	134	106	61	79
4—Prevalence	170 (83%)	95	23	14	24
5—Diagnosis	167 (81%)	93	11	7	12
6—Treatment	166 (81%)	116	8	5	7
7—Bracing	165 (80%)	70	38	23	54

### Question 1 (definition): what is idiopathic scoliosis?

**Fig. 1:**
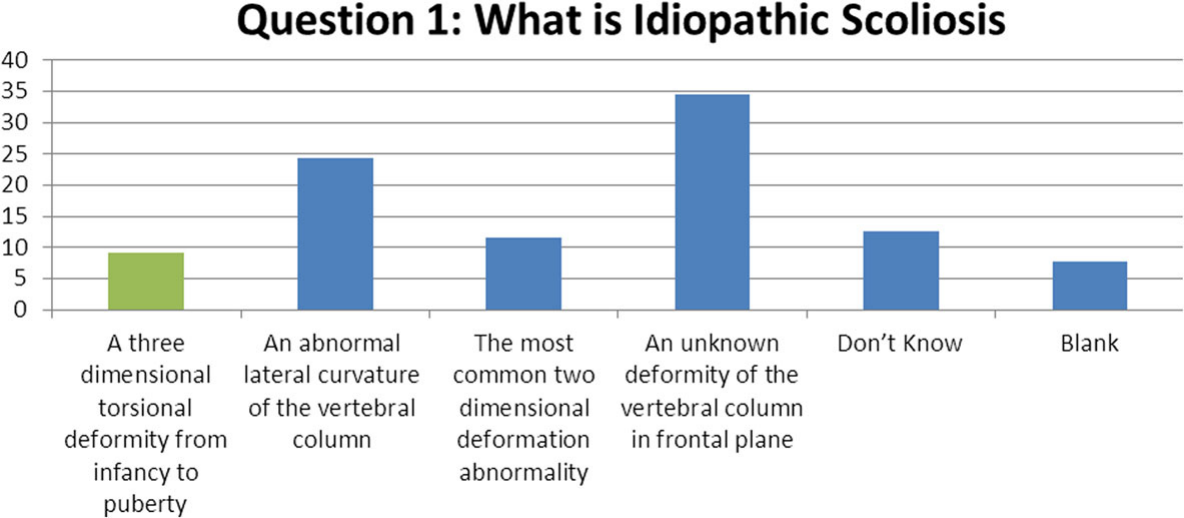
Results of question 1

Within this question, students were asked to recognise that scoliosis is a 3-dimensional deformity [6]. One hundred and forty-five of the 164 students (88%) who attempted an answer to this question selected scoliosis to either be a 2-dimensional or a lateral curvature of the spine (Fig. 1).

### Question 2 (cause): what causes idiopathic scoliosis?

**Fig. 2:**
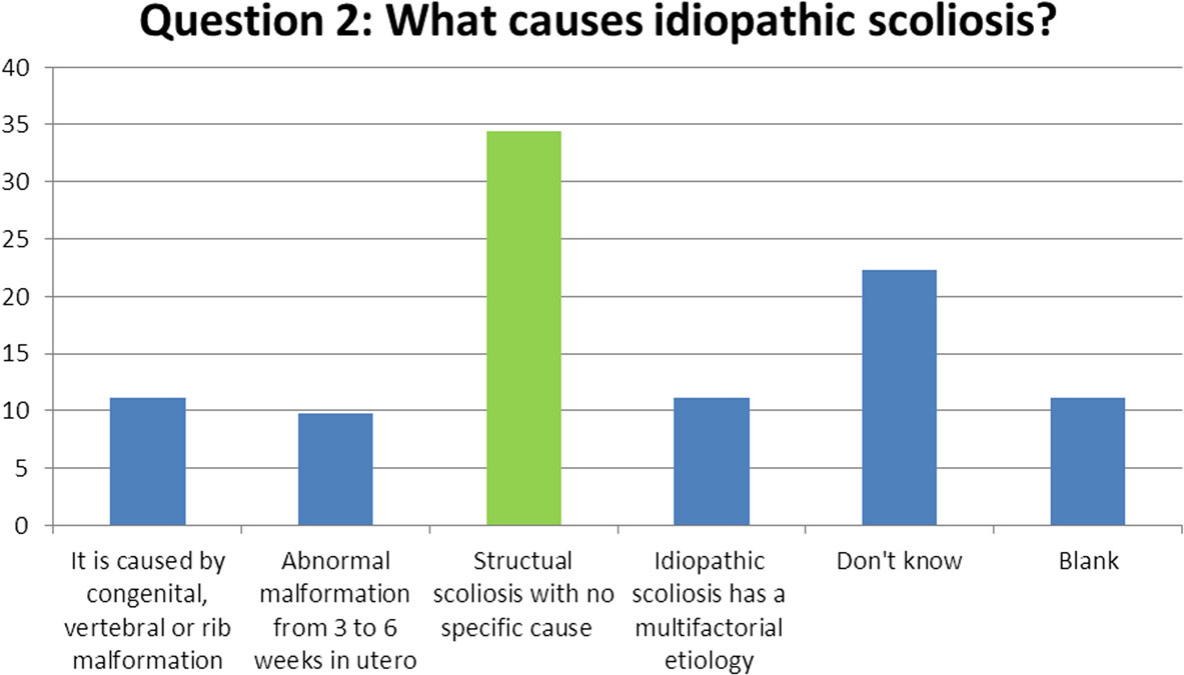
Results of question 2

When considering the potential aetiology specifically to ‘idiopathic scoliosis’, 52% of respondents highlighted that the accepted cause of IS is unknown [7] (Fig. 2).

### Question 3 (development): when does idiopathic scoliosis commonly develop?

**Fig. 3:**
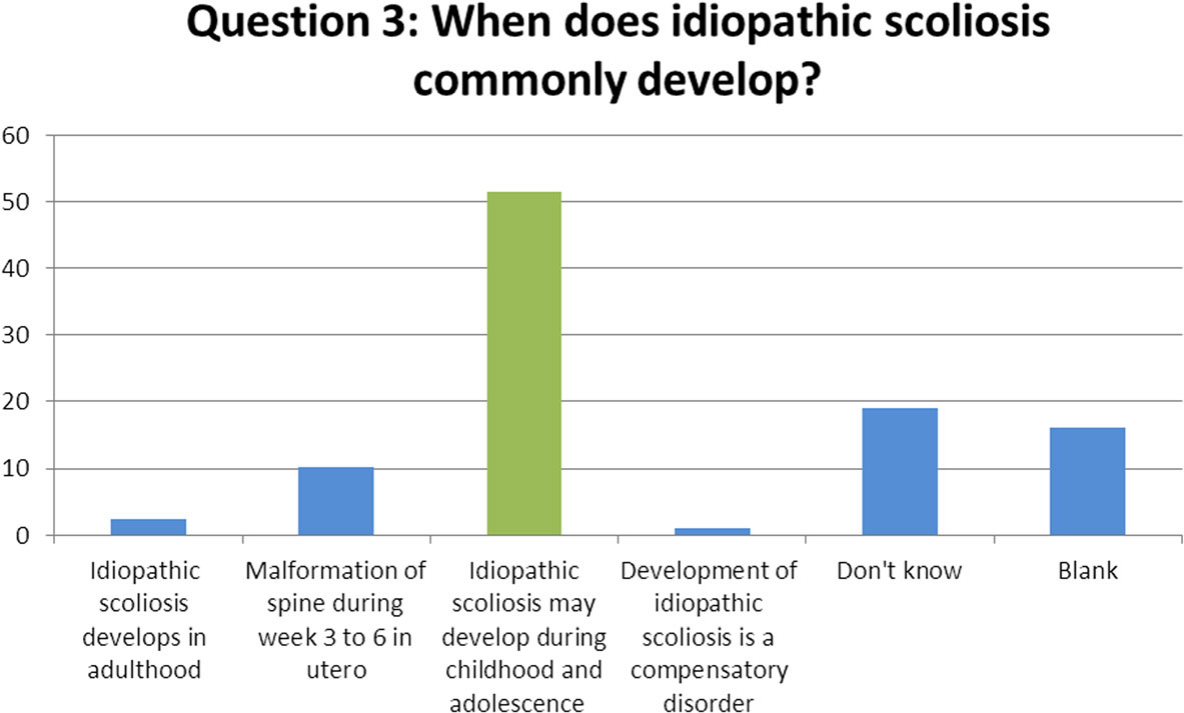
Results of question 3

One area well recognised by the students within the study was the period within which IS most commonly develops and is diagnosed. When given the options of either a period in adulthood, childhood/adolescence, in utero or as compensation to another disease, the students did correctly recognise that IS most commonly develops between a period in childhood and adolescence. Thus, they were able to identify correctly the patient group at most risk of diagnosis [8] (Fig. 3).

### Question 4 (prevalence): how prevalent is idiopathic scoliosis among patients with scoliosis?

**Fig. 4:**
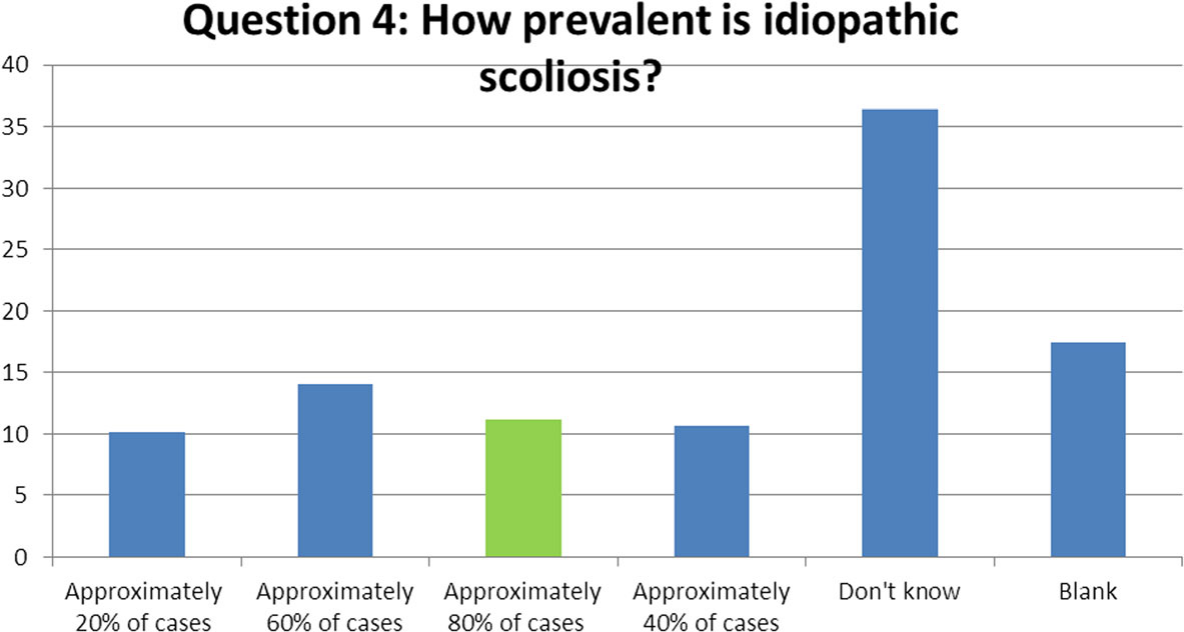
Results of question 4

Nearly a quarter (24%) of participants correctly identified the prevalence of IS within the scoliosis population as 80%. This figure is significant as it shows that when a patient is diagnosed with scoliosis, only in 20% of cases will the therapist/practitioner be able to identify a definite cause towards the development of the condition [9] (Fig. 4).

### Question 5 (diagnosis): how is the diagnosis of idiopathic scoliosis commonly confirmed?

**Fig. 5:**
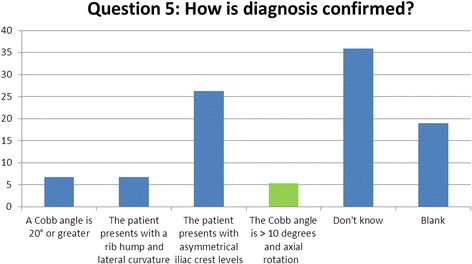
Results of question 5

In order to diagnose IS formally, a patient must present with a minimum of 10° of lateral curvature on radiography, alongside an evident and measurable amount of axial rotation [10]. The use of the Cobb angle is widely accepted as the diagnostic tool taken from radiographs, but for a conclusive diagnosis, the Cobb angle should be considered alongside a physical assessment and analysis of the structural rotation of the patient’s spine [1]**.** This strict procedure will limit any false-positive diagnostics and also provide the therapist and practitioner with more accurate information regarding the development and severity of the patient’s condition. Only 12% of respondents were able to recognise these diagnostic criteria (Fig. 5).

### Question 6 (treatment): what should the treatment of idiopathic scoliosis using therapeutic exercise include?

**Fig. 6:**
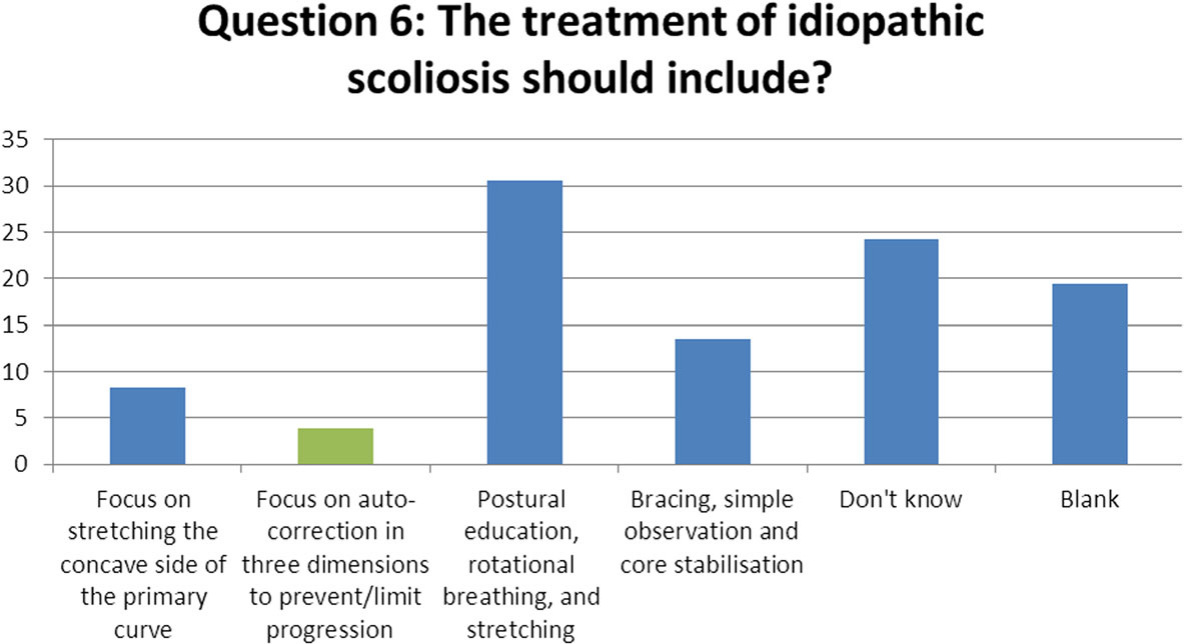
Results of question 6

There are currently, and have been historically, many different approaches to conservative management of scoliosis internationally. The wide variation in approaches and lack of availability of treatment facilities has resulted in a dilution of correct information and loss of clear management and treatment pathways in scoliosis care (Fig. 6).

With this wide gulf in different approaches, it is still largely accepted that all therapeutic exercise should be based on the recognised methods with addition of new ideas, but all based upon correction in 3-dimensions with the aim of preventing or limiting progression [11].

There is yet to be any universal approach and any self-limiting therapy such as stretching of concavities or core stabilisation exercises should always be developed with consideration to the 3-dimensional aspect of scoliosis [1].

Just 7% of respondents recognised this specific accepted view that all exercises should be based around 3-dimensional correction and aim at limiting/preventing progression.

### Question 7 (bracing): when is bracing recommended for patients with idiopathic scoliosis?

**Fig. 7:**
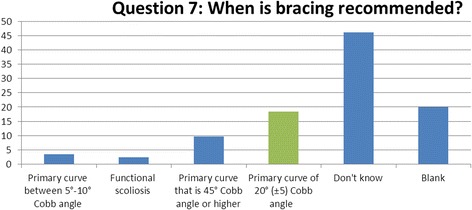
Results of question 7

Following a multi-centred, partially randomised study in 2014 [12], bracing has become an integral and decisive part of conservative management of scoliosis and its correct application is key to the benefit achieved. It is widely accepted that patients should be recommended for bracing treatment if their Cobb angle is greater than 20° and their condition is highlighted as having an elevated risk of progression, whether this be through their age, maturity level, degree of angle or physical characteristics [13] (Fig. 7).

Fifty-four percent of respondents recognised the potential use of bracing in patients with a moderate and potentially progressive curvature when offered with alternatives such as a leg length discrepancy, severe curvature (> 45°) and mild curvatures (5–10°). As bracing therapy use alongside physiotherapy has been neglected in the past, it is essential that therapists and practitioners recognise when this approach is recommended [14].

### Point of interest

**Fig. 8:**
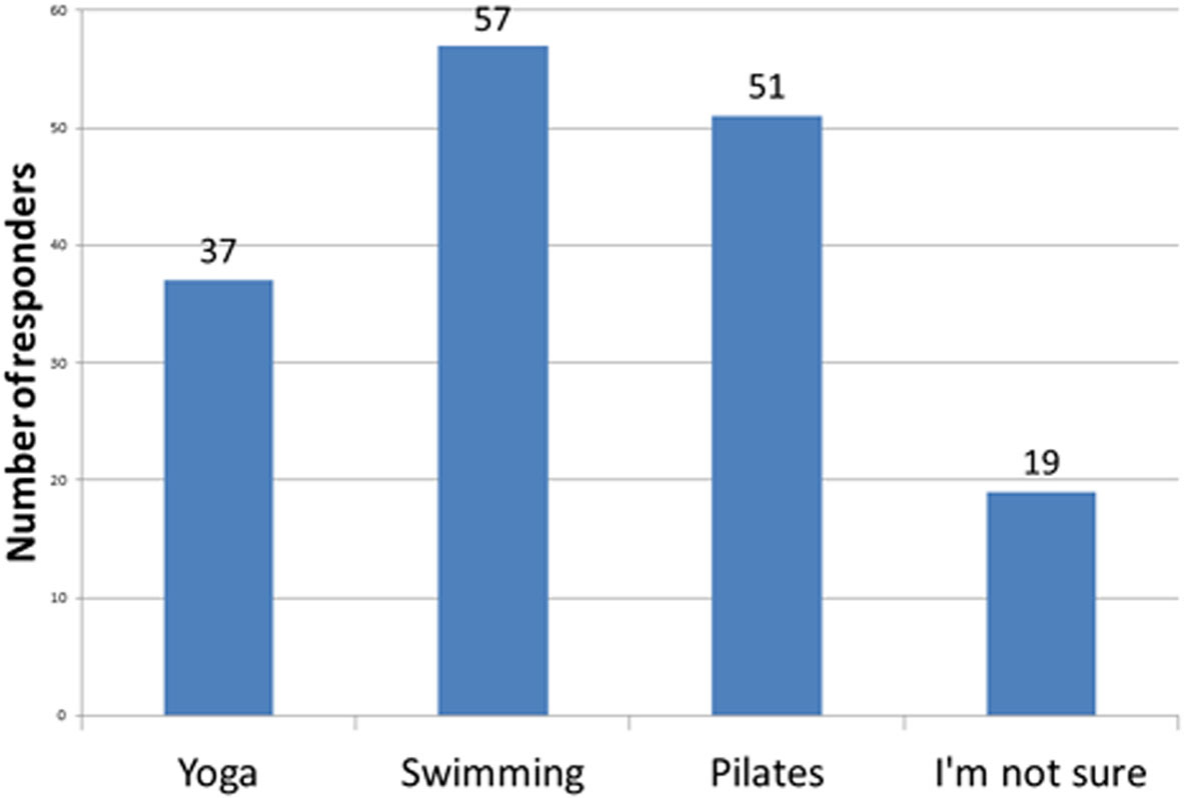
What physical activity do you think would be most beneficial for patients with idiopathic scoliosis?

**Fig. 9:**
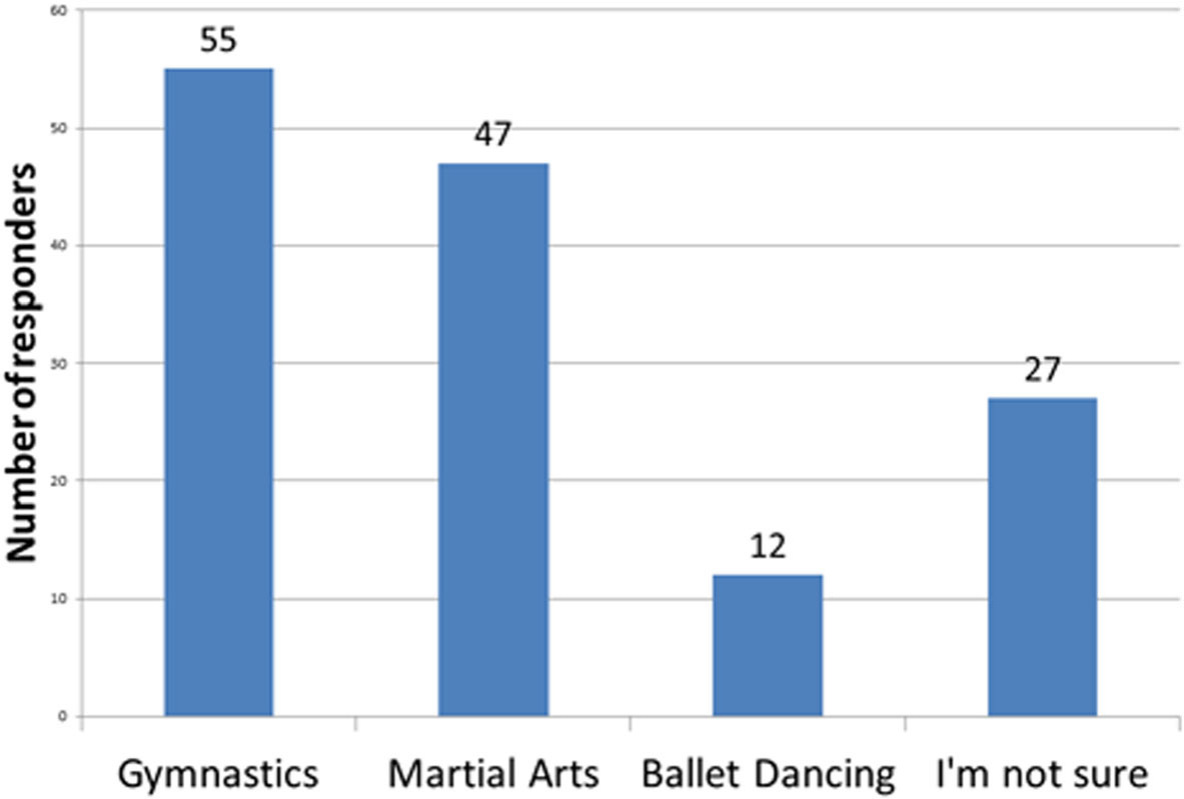
What physical activity do you think would be most harmful for patients with idiopathic scoliosis?

**Fig. 10:**
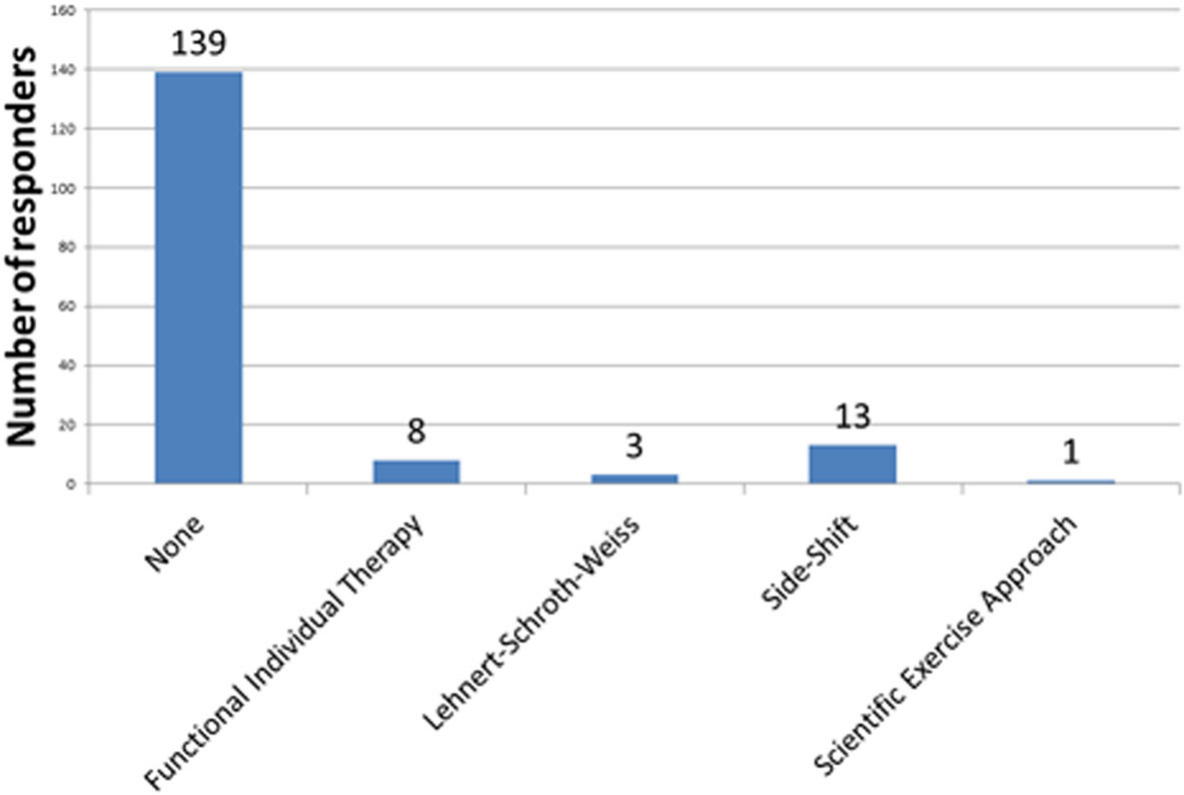
What method of conservative treatment for idiopathic scoliosis are you most familiar with?

As an opinion-based end point for the survey, the students within the study were asked three questions to highlight which physical activity they felt was most beneficial, and conversely least beneficial, for patients with scoliosis. It was also used to evaluate the participant’s knowledge of the treatment modalities highlighted as being recommended by the 2011 SOSORT guidelines. The results are highlighted in Figs. 8, 9 and 10. Subjects believed Pilates and swimming to be the most beneficial and gymnastics and martial arts to be the most detrimental and this study also demonstrates that students’ knowledge of the SOSORT recommended modalities for conservative management was very minimal. SOSORT has developed a review looking at seven different, widely accepted scoliosis schools, but 84% of students were unable to recognise any of the four most popular methods [15].

## Discussion

Our results showed a disappointing 7% of the students were able to answer more than 50% of the survey questions correctly in relation to the widely accepted guidelines on IS management. The results of the survey were particularly poor in relation to recognition of scoliosis as a 3-dimensional condition, and the application of this knowledge into their rehabilitation planning. The participants were capable of recognising the patient group and age range of patients that were likely to present with structural or developing scoliosis; however, their ability to take this a step further into diagnosis, further referral, provision of advice and prescription of exercises suitable for their patients’ scoliosis was very limited.

When comparing our results directly to previous studies on this topic, the subjects of this study performed worse in relation to their USA counterparts in all aspects except when asked, ‘What should treatment of Idiopathic Scoliosis using therapeutic exercise include?’ 6% answered correctly compared to 3% in the American study [3]. This indicates that our trainee physiotherapists in the UK are being poorly equipped to provide a satisfactory level of care to patients with scoliosis in day-to-day clinical practice. Research has shown, in Poland, physiotherapists are taught in line with the 2011 SOSORT guidelines and thus are much more familiar with the aetiology of scoliosis along with the treatment approaches available to this patient group [4].

Re-introduction of school screening has been raised in recent times, with a report in 2011 stating the reasoning behind its cessation to be very complex, involving many factors, such as the lack of accurate and reliable screening procedures, the frequency of false positives, the lack of knowledge into the natural history of scoliosis, especially in milder curves, and the evidence to support provision of efficient and effective rehabilitation protocols, again for milder curves predominantly. It reports a minimal amount of research comparing bracing or exercise therapies in relation to surgery and a lack of standardised care provision [16].

This lack of evidence, traditional non-provision of services and sparse background research into the topic has led to a severe absence in the development of education surrounding scoliosis in physiotherapists trained in the UK. This takes on even greater importance when placed in the context of the current drive towards self-referral to physiotherapy for musculoskeletal disorders. One in three clinical commissioning groups in England are now allowing for direct access to physiotherapy through self-referral [5]. This means that more and more frequently, physiotherapists will become the first point-of-contact for patients presenting with the first signs of scoliosis. Based on our results, it appears that physiotherapists in the UK are ill-equipped to provide this first point of care in relation to scoliosis and they are unlikely to be able to provide the standard of care, advice, onward referral and exercise prescription that is required to manage such a progressive and time sensitive condition.

The primary limitation to this study was the inability of the author to control the distribution of the questionnaire towards the target population. Distribution relied upon the programme lead from each individual institution to issue the questionnaire. Three leads actually refused to distribute the survey because they reported that scoliosis was not covered on their syllabus and thus their students would likely provide an unfavourable response. Without knowing the full demographic of students completing the survey and size of the population to which the questionnaire was distributed, there can be no definitive conclusion drawn on the entire UK-based student population and no comparisons can be drawn between universities.

Within the ‘Point of Interest’ questions, the respondants were asked to provide an opinion based view on physical activity with scoliosis; however, as yet there is no noted evidence to suggest whether any physical activity is beneficial for scoliosis.

Going forward, this study could be expanded to include post-graduate physiotherapists, especially focussing on the physiotherapists who are currently working within a self-referral system. This would enable analysis to be undertaken to rate the ability of these physiotherapists to identify effectively and manage scoliosis patients on a day-to-day basis within the NHS.

## Conclusion

Physiotherapy students in the UK are being let down by poor training and poor provision of information in a condition that affects 3–4% of the population. Scoliosis is a musculoskeletal condition that progresses dramatically during the adolescent years of a patient’s development, and thus should be managed with experience, knowledge and, essentially, with speed. Currently physiotherapy students are leaving university without even the most basic appreciation of scoliosis as a 3-dimensional condition and therefore are unprepared to provide accurate information in practice to patients and their families. This could potentially lead to a large number of patients being left undiagnosed or diagnosed much later, and so becoming more at risk of surgical intervention or from physical and emotional dysfunctions.

## Additional file

Additional file 1: The 10-question survey used in the study. (PDF 104 kb)

## Abbreviations

AIS: Adolescent idiopathic scoliosis
GP: General practitioner
IS: Idiopathic scoliosis
NHS: National Health Service
SOSORT: International Society on Scoliosis Orthopaedic and Rehabilitation Treatment
UK: United Kingdom
USA: United States of America
