# Supplementary material for: Current knowledge of scoliosis in physiotherapy students trained in the United Kingdom
Source: Scoliosis Spinal Disord. 2017 Sep 27;12:34. doi: 10.1186/s13013-017-0141-z (PMC5615431; doi:10.1186/s13013-017-0141-z)
Supplement: Additional file 1: — The 10-question survey used in the study. (PDF 104 kb) [file 13013_2017_141_MOESM1_ESM.pdf]

Please select ONLY ONE answer for the below questions.

1. What is idiopathic scoliosis?
    - A three dimensional torsional deformity of the spine and trunk that affects humans from infancy to after puberty.
    - An abnormal lateral curvature of the vertebral column that affects humans from infancy to after puberty.
    - The most common two dimensional deformation abnormality of the spine that has direct effects on the thoracic cage.
    - An unknown deformity of the vertebral column and trunk that results in lateral deviations of the spine in the frontal plane.
    - I don't know.

2. What causes idiopathic scoliosis?
    - It is caused by congenital, vertebral or rib malformation, and secondary to a variety of systemic or neuromuscular disorders.
    - Idiopathic scoliosis is an unknown disorder that can be attributed to a malformation of the spine during week three to six in utero.
    - Idiopathic scoliosis is a structural scoliosis for which no specific cause can be established.
    - Idiopathic scoliosis has a multifactorial aetiology that consists of shortening of a lower limb, increase in para-spinal muscle tone, or a malformation of the thoracic cage.
    - I don't know.

3. When does idiopathic scoliosis commonly develop?
    - Idiopathic scoliosis develops in adulthood between the ranges of 35 years of age and older.
    - Development of idiopathic scoliosis is attributed to a malformation of the spine during week three to six in utero.
    - Idiopathic scoliosis may develop at any time during childhood and adolescence.
    - Development of idiopathic scoliosis is a compensatory disorder that is a result from a traumatic injury or disease.
    - I don't know.

4. How prevalent is idiopathic scoliosis among patients with scoliosis?
    - Approximately 20% of cases are idiopathic scoliosis.
    - Approximately 60% of cases are idiopathic scoliosis.
    - Approximately 80% of cases are idiopathic scoliosis.
    - Approximately 40% of cases are idiopathic scoliosis.
    - I don't know.

5. How is the diagnosis of idiopathic scoliosis commonly confirmed?
    - A Cobb angle is 20° or greater.
    - The patient presents with a rib hump and a lateral curvature in the spine.
    - The patient presents with asymmetrical iliac crest levels, 20° Cobb angle, and lateral curvature in the spine.
    - The Cobb angle is ≥ 10° and axial rotation can be recognised.
    - I don't know.

6. The treatment of idiopathic scoliosis using therapeutic exercise should include:
    - Focus on stretching the concave side of the primary curve and strengthening the convex side of the primary curve.
    - The adaptation of old techniques and the addition of new forms that focus on auto-correction in three dimensions to prevent/limit progression.
    - Postural education, rotational breathing, and stretching have been shown to be the gold standard in research when considering treatment of idiopathic scoliosis.
    - Conservative care that includes bracing, simple observation, and core stabilization exercises
    - I don't know.

7. When is bracing recommended for patients with idiopathic scoliosis?
    - Patients that present with a primary curve between the ranges of 5°-10° Cobb angle should be recommended for scoliosis bracing.
    - Bracing is recommended for patients that have been diagnosed with functional scoliosis that is secondary to a leg length discrepancy of 6mm or greater.
    - Patients that present with a primary curve that is 45° Cobb angle or higher should be recommended for scoliosis bracing.
    - Bracing is recommended for patients with a 20° (±5) Cobb angle that have an elevated risk of progressing.
    - I don't know.

8. What physical activity do you think would be most beneficial to patients with idiopathic scoliosis?
    - Swimming
    - Yoga
    - Martial Arts
    - Jogging
    - I don't know.

9. What physical activity do you think would be most harmful to patients with idiopathic scoliosis?
    - Gymnastics
    - Ballet Dancing
    - Martial Arts
    - Cycling
    - I don't know.

10. What method of conservative treatment of idiopathic scoliosis are you most familiar with?
    - Lehnert-Schroth-Weiss
    - BSPTS
    - FITS
    - SEAS
    - Dobomed
    - Lyon method
    - Side shift
    - None.
